# Molecular Determinants of Ebola Virus Virulence in Mice

**DOI:** 10.1371/journal.ppat.0020073

**Published:** 2006-07-21

**Authors:** Hideki Ebihara, Ayato Takada, Darwyn Kobasa, Steven Jones, Gabriele Neumann, Steven Theriault, Mike Bray, Heinz Feldmann, Yoshihiro Kawaoka

**Affiliations:** 1 Institute of Medical Science, University of Tokyo, Tokyo, Japan; 2 CREST, Japan Science and Technology Agency, Saitama, Japan; 3 Special Pathogens Program, National Microbiology Laboratory, Public Health Agency of Canada, Winnipeg, Manitoba, Canada; 4 Hokkaido University Research Center for Zoonosis Control, Sapporo, Japan; 5 Respiratory Viruses, National Microbiology Laboratory, Public Health Agency of Canada, Winnipeg, Manitoba, Canada; 6 Department of Immunology, University of Manitoba, Winnipeg, Manitoba, Canada; 7 Department of Pathobiological Sciences, School of Veterinary Medicine, University of Wisconsin-Madison, Madison, Wisconsin, United States of America; 8 Department of Medical Microbiology, University of Manitoba, Winnipeg, Manitoba, Canada; 9 Biodefense Clinical Research, Office of Clinical Research, Office of the Director, National Institute of Allergy and Infectious Diseases, National Institutes of Health, Bethesda, Maryland, United States of America; 10 International Research Center for Infectious Diseases, Tokyo, Japan; Robarts Research Institute, Canada

## Abstract

*Zaire ebolavirus* (ZEBOV) causes severe hemorrhagic fever in humans and nonhuman primates, with fatality rates in humans of up to 90%. The molecular basis for the extreme virulence of ZEBOV remains elusive. While adult mice resist ZEBOV infection, the Mayinga strain of the virus has been adapted to cause lethal infection in these animals. To understand the pathogenesis underlying the extreme virulence of Ebola virus (EBOV), here we identified the mutations responsible for the acquisition of the high virulence of the adapted Mayinga strain in mice, by using reverse genetics. We found that mutations in viral protein 24 and in the nucleoprotein were primarily responsible for the acquisition of high virulence. Moreover, the role of these proteins in virulence correlated with their ability to evade type I interferon-stimulated antiviral responses. These findings suggest a critical role for overcoming the interferon-induced antiviral state in the pathogenicity of EBOV and offer new insights into the pathogenesis of EBOV infection.

## Synopsis

*Zaire ebolavirus* causes severe hemorrhagic fever in humans with up to 90% case-fatality rates. Currently, there are no vaccines or specific therapeutic interventions available for this devastating viral disease due, at least in part, to a lack of knowledge regarding the molecular basis of virulence for this extremely pathogenic agent. While adult mice resist wild-type *Zaire ebolavirus* infection, the virus has recently been adapted to cause lethal infection in mice. In order to understand the pathogenesis underlying *Zaire ebolavirus* infection, the authors identified the mutations responsible for the acquisition of virulence in mice, using reverse genetics technology, which allows the generation of genetically altered mutant viruses from cloned cDNA. By testing the virulence of mutant viruses, two viral proteins, viral protein 24 and the nucleoprotein, were found to be primarily responsible for the acquisition of virulence in mice. Moreover, the role of these proteins in virulence correlated with their ability to confer resistance to interferon-stimulated antiviral responses in mouse cells. These findings suggest a critical role of these proteins in overcoming the interferon-induced antiviral state in the pathogenicity of *Zaire ebolavirus* and offer new insights into the pathogenesis of *Zaire ebolavirus* infection.

## Introduction

*Zaire ebolavirus* (ZEBOV), a member of the family *Filoviridae*, genus *Ebolavirus,* causes severe hemorrhagic fever in humans and nonhuman primates (NHPs). Case-fatality rates for ZEBOV infection in humans are the highest among known viral hemorrhagic fevers, ranging from 70% to 90% [1–3]. On the basis of in vitro data, three Ebola virus (EBOV) proteins, the glycoprotein (GP) [4–6], the membrane-associated viral protein (VP) 24 [7,8], and VP35 [9,10], a component of the replication complex, are thought to play key roles in EBOV pathogenicity. The GP, which mediates viral entry, is a major determinant of viral tropism and may be cytotoxic, although a recent report has challenged the notion of GP's cytotoxicity [4–6,11]. VP24 and VP35 are known as type I interferon (IFN) antagonists and interfere with the type I IFN-mediated antiviral response in vitro [7,9,10]. However, the role of these proteins in viral pathogenicity has not been determined in vivo.

Three animal models, NHPs, guinea pigs, and mice, have been used to study EBOV pathogenesis [12–14]. Generally, filoviruses do not kill adult immunocompetent rodents, although some strains have been shown to cause lethal infections in newborn mice [14]. Bray et al. [14] adapted ZEBOV to progressively older BALB/c mice and thereby established a lethal model in adult immunocompetent mice. Infection of mice with mouse-adapted virus (MA-ZEBOV) involves primary target cells of the mononuclear phagocytic system, namely monocytes, macrophages, and dendritic cells, as well as target organs (spleen, lymph nodes, and liver), as seen in humans and NHPs, resulting in a disease comparable to that observed in the latter animals [2,15–17]. Although MA-ZEBOV–infected mice do not exhibit coagulation abnormalities, a hallmark of EBOV infection in humans and NHPs, this is understandable given that coagulopathy is not generally seen in mouse models for acute viral infections [15,18]. Thus, this mouse model may not exactly mirror all aspects of human Ebola hemorrhagic fever; however, it does provide a relevant and convenient animal model with which to study aspects of pathogenicity and host immune response in vivo [19–21].

The adaptation of ZEBOV to adult mice resulted in a number of nucleotide changes in both the coding and noncoding regions (NCRs) of the virus genome [22]. To identify the molecular features that determine EBOV virulence in mice, here, we exploited a reverse genetics system to generate infectious ZEBOV entirely from cloned cDNA [23] and artificially generate recombinant viruses possessing various combinations of wild-type and mouse-adapted genes. The virulence of these recombinant viruses was then tested in adult immunocompetent mice.

## Results

### Construction and Generation of Recombinant MA-ZEBOV Mutants from cDNAs

**Figure 1:**
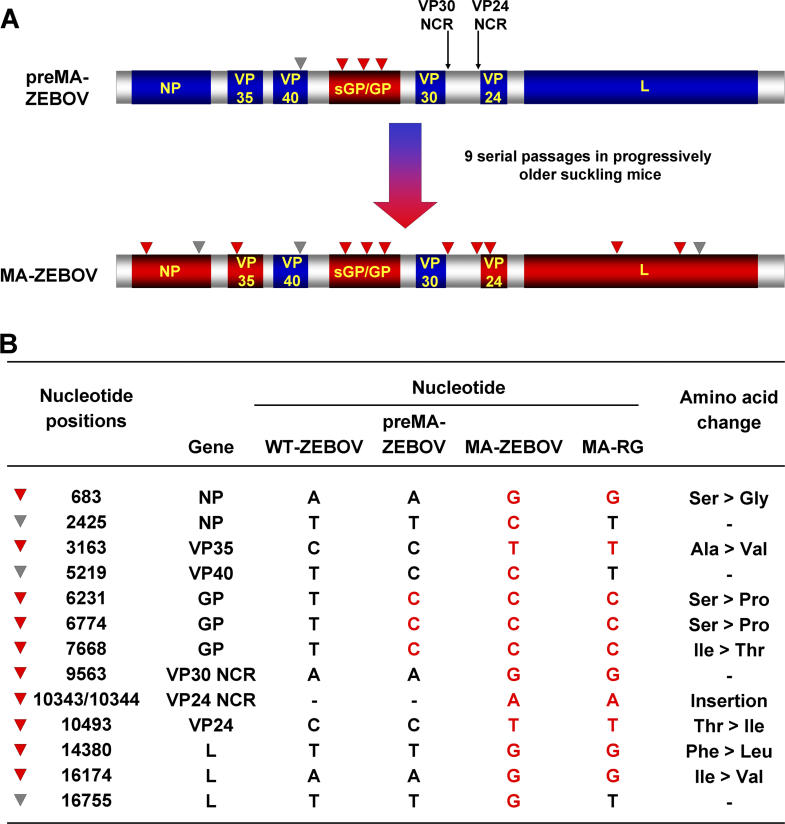
Molecular Differences among ZEBOV Mouse-Adapted Variants (A) Comparison of ZEBOV variants. Pre–MA-ZEBOV differs from wild-type ZEBOV (WT-ZEBOV) by three amino acids in the GP (red triangles) and a silent mutation in the ORF of the VP40 gene (gray triangle). Serial passage of pre–MA-ZEBOV in progressively older mice yielded MA-ZEBOV [14], which contains coding changes in the NP, VP35, VP24, and polymerase (L) (all shown in red triangles). Two nucleotide changes localize to the NCRs at the 3′ end of the VP30 and the 5′ end of the VP24 gene (red triangles); the remaining three modifications in the NP, VP40, and L ORFs are silent (gray triangles). (B) Nucleotide and amino acid differences among WT-ZEBOV, pre–MA-ZEBOV, MA-ZEBOV, and MA-RG. The nucleotide changes in GP of pre–MA-ZEBOV, compared to WT-ZEBOV, as well as the changes acquired during adaptation of pre–MA-ZEBOV in mice are shown in red. −, no change.

The ZEBOV variant that served as the starting point for adaptation in mice (referred to as precursor mouse-adapted virus [pre–MA-ZEBOV]) differed from the published sequence of the wild-type ZEBOV (WT-ZEBOV), strain Mayinga, in four nucleotide positions. These mutations may have been acquired during three consecutive passages in the brains of newborn mice and/or two passages in Vero E6 cells [14]. Three of these mutations resulted in amino acid changes in the glycoprotein (GP), while the fourth created a silent mutation in the open reading frame (ORF) encoding VP40 (Figure 1A and 1B). Serial passage of pre–MA-ZEBOV in progressively older suckling mice yielded the fully mouse-adapted variant (MA-ZEBOV), which possessed nine additional nucleotide changes. Nucleotide substitutions leading to amino acid changes were found in VP35, VP24, nucleoprotein (NP) (one amino acid change each), and the polymerase protein L (two amino acid changes) (Figure 1A and 1B). An additional two nucleotide changes were found in NCRs (Figure 1A and 1B), which may affect replication and/or transcription efficiencies. The remaining two nucleotide modifications, in the NP and L genes, were silent.

**Figure 2:**
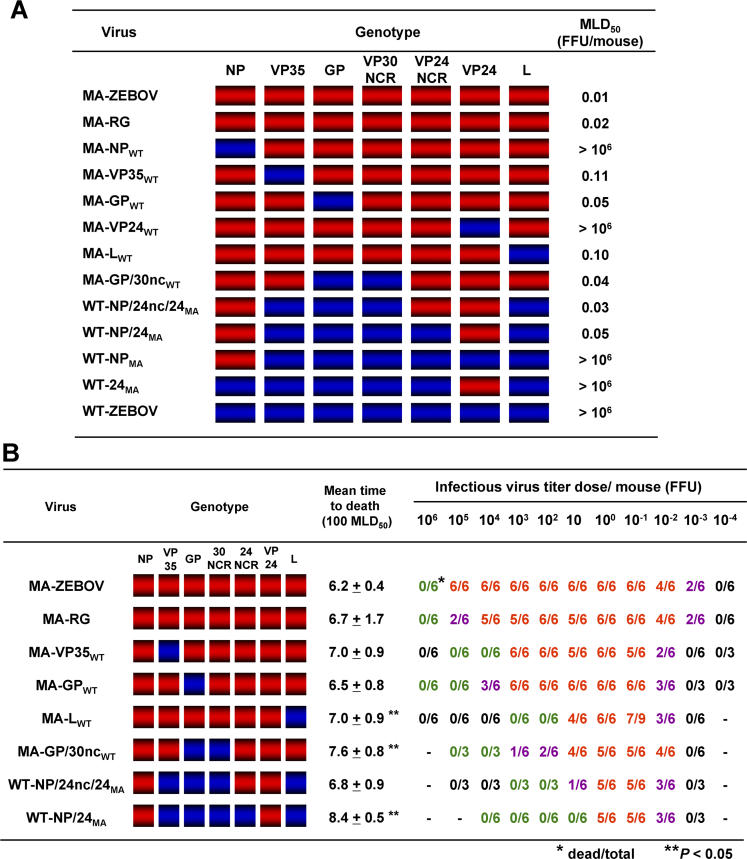
Genetic Determinants of Virulence in Adult Mice (A) Determination of MLD_50_ values. Bars indicate the genotype of the viral gene: MA-ZEBOV (red), WT-ZEBOV (blue). The MLD_50_ values of recombinant viruses were determined by i.p. inoculation of mice (three to six per group) with serial 10-fold dilutions of virus stock and then monitoring of survival rates. Experiments were carried out in duplicate. (B) Determination of the mean time to death and dose ranges causing morbidity/mortality. The mean time to death of mice inoculated with 10 FFU (approximately 1,000 MLD_50_ for MA-ZEBOV) are indicated. Differences in the mean time to death for mice infected with various mutants, compared to that of mice infected with MA-ZEBOV, were considered significant when the *p-*value was <0.05. The dose range for morbidity/mortality was determined by inoculating groups of six to nine mice with the indicated amounts of viruses and monitoring the mice for weight loss and time to death. Survival numbers (dead/total) are color-coded to indicate the severity of infection in infected mice: no disease (black), illness without mortality (green), less than or equal to 50% mortality (purple), greater than 50% mortality (red). *Dead/total. **p < 0.05.

Using a reverse genetics system to create ZEBOV from plasmid DNAs [23], we generated MA-RG (mouse-adapted reverse genetics virus) by introducing all but three mutations into the backbone of WT-ZEBOV (Figure 1B). MA-RG differed from MA-ZEBOV by the three silent mutations in the NP, VP40, and L ORFs that were introduced during adaptation in mice (Figure 1B). We also generated a variety of recombinant viruses containing various subsets of the mutations found in MA-ZEBOV (depicted in Figure 2A) to identify those mutations responsible for mouse adaptation.

### The VP24 and NP Genes Determine ZEBOV Virulence in Mice

To identify the genetic determinants of ZEBOV virulence in mice, we inoculated groups of BALB/c mice (5 to 6 wk old; three to six per group) intraperitoneally (i.p.) with 10 focus-forming units (FFU) of WT-ZEBOV, the original mouse-adapted virus (MA-ZEBOV), its counterpart generated using reverse genetics (MA-RG), or viruses containing subsets of the mutations found in MA-ZEBOV (Figure 2A). This dose (10 FFU) corresponded to 1,000 MLD_50_ (the amount of virus required to kill 50% of the mice) for MA-ZEBOV. As expected, infection of mice with WT-ZEBOV did not produce a lethal infection or any clinical symptoms, unlike infection with either MA-ZEBOV or MA-RG. Interestingly, viruses containing the wild-type NP or VP24 genes in the background of MA-ZEBOV (MA-NP_WT_, MA-24_WT_) did not cause lethal infection or clinical symptoms in mice, suggesting a critical role for mutations in these proteins with respect to the virulence of MA-ZEBOV in mice. In contrast, substitution of the VP35, GP, and/or L genes of MA-RG with those of WT-ZEBOV did not dramatically reduce the virulence of the virus (Figure 2A). Determination of the MLD_50_, by i.p. inoculation of mice with 10-fold serial dilutions of viruses, revealed almost identical MLD_50_ values for all of these recombinant viruses with few exceptions (Figure 2A). We, therefore, tested the inverse genotypes by introducing mouse-adapted NP and VP24 genes, separately and in combination, into the background of WT-ZEBOV. Only WT-NP/24_MA_ resulted in a lethal phenotype; neither of the single gene introductions (WT-NP_MA_, WT-24_MA_) caused disease in mice. These findings identified the specific amino acid changes in NP and VP24 that are the critical determinants of ZEBOV virulence in adult mice.

### Multiple Mutations in MA-ZEBOV Contribute to Enhanced Virulence in Mice

All mice infected with virulent viruses displayed the same clinical symptoms (ruffled fur, decreased activity, and weight loss), but notable differences were observed in the mean time to death and in the dose ranges that caused morbidity or mortality (Figure 2B). Infection with MA-ZEBOV resulted in the shortest mean time to death, with lethal infection induced by a broad range of inoculum doses (10^5^ to 10^−3^ FFU/mouse). The artificially generated MA-RG virus was slightly attenuated, suggesting that the silent mutations in the NP, VP40, and L genes, by which this mutant differs from MA-ZEBOV, may make a minor contribution to virulence.

Similarly, the introduction of the mouse-adapted NP and VP24 genes into the background of WT-ZEBOV (WT-NP/24_MA_) did not bestow on WT-ZEBOV the fully pathogenic phenotype, as demonstrated by a prolonged time to death and a narrower dose range causing mortality than that of MA-ZEBOV. Insertion of the mutation into the 5′ NCR of the VP24 gene of WT-NP/24_ MA_ (i.e., WT-NP/24nc/24_MA_) reduced the time to death but did not appreciably expand the dose range for morbidity/mortality, indicating a rather minor contribution of this mutation to virulence in mice. In contrast, the nucleotide substitution in the 3′ NCR of VP30 caused a more appreciable effect on mouse virulence (compare MA-GP_WT_ and MA-GP/30nc_WT_, which differ only in the VP30 NCR).

Single gene replacements of the mouse-adapted VP35, GP, or L with their wild-type counterparts (MA-VP35_WT_, MA-GP_WT_ MA-L_WT_) led to different degrees of attenuation, indicating that all of the mutations contributed to some extent to the virulence of MA-ZEBOV in adult mice. Of these mutant viruses, MA-L_WT_ showed the most pronounced attenuation, indicating that the two amino acid changes in the L protein of MA-ZEBOV are more critical for virulence in mice than those in the VP35 or GP proteins.

### Growth Characteristics in Mouse Organs

**Figure 3:**
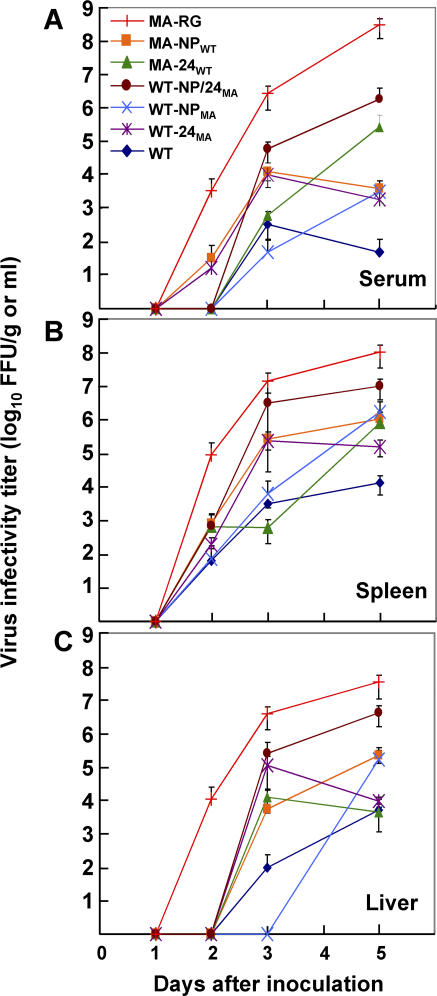
Growth Characteristics of Recombinant Viruses in Mice Groups of 12 mice were inoculated i.p. with 5 FFU (approximately 500 LD_50_ values for MA-ZEBOV) of representative viruses. On days 1, 2, 3, and 5 postinoculation, selected organs were removed from three infected animals per group. Virus titers in serum (A), spleen (B) and liver (C) were determined in Vero E6 cells by using a focus-forming assay [36].

We next compared the growth characteristics of representative mutant viruses in mice infected with 5 FFU of virus by examining viral titers in the serum (Figure 3A) and in two target organs of EBOV, the spleen and liver (Figure 3B and 3C). As expected, WT-ZEBOV replicated poorly in mice, whereas the virus titers of MA-RG were the highest in all organs tested, again demonstrating that the full set of mutations is required to establish the fully pathogenic phenotype. Efficient virus replication required both the mouse-adapted NP and VP24 genes (compare WT-NP/24_MA_ with WT-NP_MA_ and WT-VP24_MA_), a finding that correlated well with virulence, as measured by lethality (Figure 2A and 2B). Likewise, the introduction of either the wild-type NP or VP24 gene into the background of MA-ZEBOV attenuated the resulting recombinant viruses (MA-NP_WT_, MA-24_WT_), further emphasizing the critical role of these mutations for viral replication in mice.

### Do Mutations in VP24 and NP Affect Virus Sensitivity to IFN?

**Figure 4:**
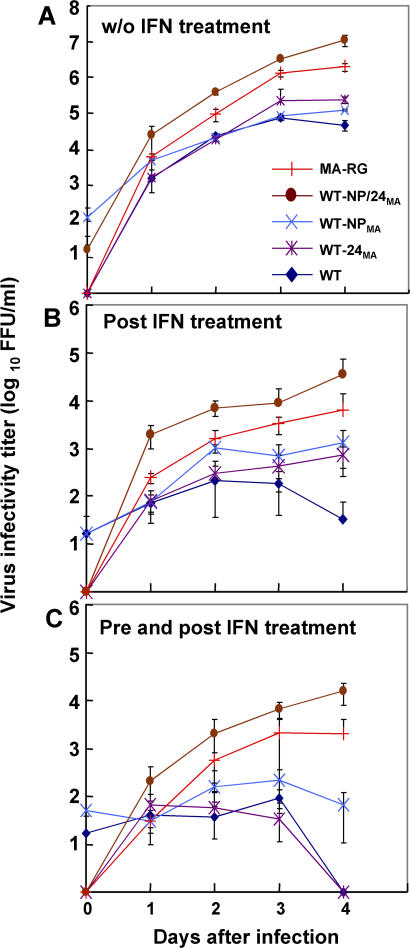
Effect of Murine Type I IFNs on Recombinant Virus Replication in Mouse Macrophages RAW 264.7 cells (mouse peritoneal macrophage-derived cell line) were infected with a multiplicity of infection of 0.05. Cells were untreated (A), treated with murine IFN-α/β (500 units/ml) 2 h postinfection (B), or treated with murine IFN-α/β (500 units/ml) 12 h prior to and again 2 h after virus adsorption (C). Supernatants were collected on days 0, 1, 2, 3, and 4 postinfection and titrated by use of a focus-forming unit assay in Vero E6 cells [36].

The suppression of viral multiplication by type I IFN-mediated antiviral responses has been demonstrated in many viruses [24], including EBOV [25]. In fact, it is abrogation of the type I IFN (IFN-α/β) system that makes adult mice susceptible to EBOV infection [25]. We and others have identified EBOV VP24 as being instrumental in this function (i.e., in inhibiting IFN signaling) [7,26]. To determine whether the amino acid changes in the VP24 and NP proteins of MA-ZEBOV facilitate evasion of the type I IFN-induced antiviral response, we compared virus growth of selected recombinant viruses in a mouse peritoneal macrophage cell line (RAW 264.7 cells) in the absence or presence of murine IFN-α/β (Figure 4). WT-ZEBOV grew in nonstimulated cells, albeit to lower titers compared to the growth of the other viruses tested (Figure 4A); however, in cells stimulated with IFN at 2 h postinfection (Figure 4B) or 12 h prior to and 2 h after infection (Figure 4C), virus replication was severely reduced. In contrast, MA-RG grew to much higher titers even in IFN-stimulated cells, suggesting that its virulence in mice is linked to its ability to counteract IFN-induced antiviral responses. This ability clearly correlated with the mutations in the mouse-adapted VP24 and NP genes. In fact, WT-NP/VP24_MA_ grew more efficiently in IFN-stimulated cells than did MA-RG, for unknown reasons. In cells that were stimulated with IFN postinfection, WT-NP_MA_ or WT-VP24_MA_ grew better than WT-ZEBOV. In contrast, WT-VP24_MA_, but not WT-NP_MA_, failed to replicate efficiently in cells treated with IFN prior to and after infection. These data indicate that the ability to counteract IFN-induced antiviral responses is responsible for the high virulence of MA-ZEBOV in this animal model and that IFN evasion may be critical for EBOV virulence in other animal models, as well as in humans. In addition, our findings show that while both mouse-adapted VP24 and NP provide the virus with the ability to counteract the IFN-induced antiviral responses, NP may be more critical for this effect in vitro. The concerted actions of both mutations, however, appear necessary for evading IFN-induced antiviral responses in vivo.

## Discussion

In this study, we have identified molecular determinants of ZEBOV virulence in mice by using reverse genetics. We found VP24 and NP to be the primary determinants for adaptation of WT-ZEBOV in mice and found a correlation between virulence and the ability of the virus to evade the type I IFN-induced antiviral response. The ability to overcome the IFN-induced antiviral response is, therefore, a critical event in the pathogenesis of ZEBOV infection in mice and possibly in humans. Moreover, mutations in other viral proteins and NCRs also contribute to the virulent phenotype, indicating that virulence is a multifactor trait.

Recent in vitro studies suggest that VP24 functions as a type I IFN antagonist [7,26], but the significance of this finding had not been addressed in vivo until now. Here, we have demonstrated that single amino acid modifications in VP24 and NP of MA-ZEBOV are critical for virus evasion of IFN-induced antiviral responses. NP has not previously been considered as a potential IFN antagonist. However, given that it interacts with VP24 in the formation of nucleocapsids [27,28], it is not unreasonable to imagine that it may act as an enhancer and/or stabilizer of VP24 functions. Nevertheless, since the NP mutation acquired during mouse adaptation of WT-ZEBOV alone allowed the recombinant virus to replicate in IFN-treated cells (see Figure 4C), NP likely plays a more direct role in the evasion of the IFN-induced antiviral response. Interestingly, WT-NP/VP24_MA_ grew more efficiently than MA-RG in IFN-stimulated cells, in contrast to our in vivo results. For this reason, it seems likely that the mutations in VP24 and NP are critical for resistance to IFN-induced antiviral responses, while the remaining mutations acquired during adaptation of WT-ZEBOV facilitate efficient virus replication and/or spread in mice despite their attenuated phenotype in cell culture systems.

VP35 and GP have previously been linked to EBOV pathogenicity [4–6,9,10]. VP35 is an IFN antagonist that interferes with type I IFN synthesis by inhibiting IRF-3 (interferon regulatory factor 3) activation, a necessary step for the transcription of IFN genes [9,10]. Although the mutation found in the mouse-adapted VP35 protein was not responsible for the enhanced virulence of MA-ZEBOV (Figure 2B), this does not necessarily indicate that VP35-mediated regulation of IFN levels does not play a part in the pathogenicity of EBOV. This activity may still be important for EBOV to achieve high virulence. Likewise, GP is cytotoxic and is, therefore, thought to contribute to viral pathogenicity [5,29,30]. However, a recent report suggested that this cytotoxicity originated from overexpression of GP in cells [11]. As with VP35, the lack of an adaptive mutation in the GP of MA-ZEBOV does not necessarily diminish the role of GP in viral pathogenicity.

Interestingly, the adaptation of ZEBOV in guinea pigs also resulted in amino acid changes in NP, VP24, and L and in a nucleotide substitution in the VP30 NCR [8]. Although these mutations differed in their amino acid positions from those in MA-ZEBOV, it seems likely that they serve a similar role in adaptation and also function by counteracting innate antiviral responses [31,32]. As with mice, additional mutations (e.g., in L and the VP30 NCR) likely contribute to virulence by affecting viral transcription/replication.

The mouse model using MA-ZEBOV does not exactly mirror all aspects of human Ebola hemorrhagic fever [15,18]. Thus, determinants for virulence of ZEBOV may differ between mouse and primate models. However, since ZEBOV is naturally lethal to primates, but not to mice, this model provided an opportunity to decipher the roles of viral proteins in expression of high virulence in a host (i.e., mice). Our studies showed that ZEBOV VP24 and NP are inadequate for the expression of high virulence in mice, but upon mutation, optimally expressed this property. Thus, it is possible that these viral proteins play an important role in expression of high virulence in primates. Likewise, the lack of VP35 mutations correlating with pathogenicity in mice is interesting, indicating that ZEBOV VP35 optimally functions in both primates and mice without additional mutation in an IFN pathway. Since MA-ZEBOV is attenuated in NHPs [18], one or more genes into which mutations were introduced during mouse adaptation of ZEBOV likely play a role in virulence in primates. Therefore, it will be interesting to examine the virulence of selected recombinant mouse-adapted variants in NHPs. Such studies will provide us with valuable information for understanding ZEBOV pathogenesis in humans.

Of note, we observed resistance in mice to infection with high infectious doses of MA-ZEBOV (Figure 2B). A similar finding has been observed in a mouse model for rabies virus [33]. The most likely explanation for this finding is that inoculation with high doses of virus causes a rapid stimulation of the innate immune response before virus replication or spread can occur. This topic presents an attractive research subject that may lead to control measures for EBOV infections through immunologic modulation of host responses to viral infection.

In conclusion, the combination of reverse genetics technology [34] and a small-animal model has allowed us to gain valuable insights into EBOV pathogenesis. Understanding the molecular basis of mouse adaptation of ZEBOV will likely lead to the identification of viral genetic determinants of EBOV virulence and to the elucidation of the roles of specific viral proteins in the pathogenic process. A more detailed molecular understanding of virulence and the host responses will also be crucial to improving our ability to control EBOV infections in the future.

## Materials and Methods

### Cells.

Vero E6 (African green monkey kidney) cells, 293T (human embryonic kidney) cells, and RAW 264.7 cells (mouse peritoneal macrophages) were grown in DMEM supplemented with 10% FBS, 2 mM l-glutamine, and penicillin (100 U/ml)-streptomycin (100 μg/ml). Cells were incubated at 37 °C in 5% CO_2_.

### Generation of mutant Ebola viruses.

Starting with a cDNA clone encoding WT-ZEBOV, strain Mayinga [23], we introduced stepwise mutations to reproduce MA-ZEBOV, using PCR-based mutagenesis. The resulting plasmids, which are flanked by T7 RNA polymerase promoter and ribozyme sequences [23], were cotransfected into a mixed culture of Vero E6 and 293T cells, together with helper plasmids for the expression of T7 RNA polymerase and EBOV NP, VP35, VP30, and L (required components of the viral replication complex), following established protocols [23,35]. All viruses were amplified once in Vero E6 cells. DMEM supplemented with 2% FBS was used to prepare virus stocks.

Virus infectivity titers (FFU) were obtained by counting the number of infected cell foci detected by use of an indirect immunofluorescent antibody assay, as previously described [36]. EBOV antigen-positive foci were detected with a rabbit polyclonal anti-VP40 antibody and a goat anti-rabbit IgG-FITC conjugate [37].

### Animal experiments.

Five- to 6-wk-old female BALB/c mice were obtained from a commercial supplier (Charles River Laboratories, Wilmington, Massachusetts, United States). All mice were housed in microisolator cages and allowed to acclimatize for 5 days prior to use in experiments.

To assay virulence, groups of three to six mice were each inoculated i.p. at two different sites with 10 FFU of virus in 0.1 ml of DMEM. Following infection, mice were observed daily for clinical symptoms and their weights were recorded for 11 d postinoculation. All surviving animals were observed for at least 21 d (three times the average duration of survival of the control animals).

The MLD_50_ was determined by i.p. inoculation of mice (three to six per group) with serial 10-fold dilutions of virus and monitoring of the survival rates.

To assess virus growth characteristics in mice, groups of 12 animals were inoculated i.p. with 5 FFU of virus (corresponds to approximately 500 MLD_50_ for MA-ZEBOV). On days 1, 2, 3, and 5 postinfection, spleen, liver, and blood were collected from three infected mice, and the spleen and liver samples were homogenized. Viral infectivity titers were determined by use of a focus-forming assay in Vero E6 cells [36].

All work with live EBOV was performed in the BSL-4 laboratory of the National Microbiology Laboratory of the Public Health Agency of Canada. All animal experiments were performed in accordance with approved animal use documents and according to the guidelines of the Canadian Council on Animal Care.

### Replication kinetics in IFN-stimulated murine cells.

RAW 264.7 cells were infected with the respective viruses at a multiplicity of infection of 0.05. The cells were either left untreated or treated with 500 units/ml murine IFN-α/β 2 h postinfection, or 12 h prior to infection and again 2 h postinfection. For all samples, virus titers in the supernatants were determined on days 0, 1, 2, 3, and 4 postinfection by use of the focus-forming assay in Vero E6 cells [36]. The first sample (day 0) was collected after the virus had adsorbed and the cell monolayer had been washed three times.

### Statistical analyses.

All virus titers in the growth kinetics experiments are shown as the mean ± SEM. The *p-*values in Figure 2B were calculated by using the Student's *t*-test (two-tailed distribution, two-sample unequal variance). *p* < 0.05 was considered to indicate statistical significance.

## Supporting Information

### Accession Numbers

The GenBank accession numbers for the genes mentioned in this paper are WT-ZEBOV, strain Mayinga (AF086833) and MA-ZEBOV variant (AF499101).

## Abbreviations

EBOV: Ebola virus
FFU: focus-forming units
IFN: interferon
i.p.: intraperitoneal
MA-RG: mouse-adapted reverse genetics virus
MA-ZEBOV: mouse-adapted virus
MLD_50_: amount of virus required to kill 50% of the mice
NCR: noncoding region
NHP: nonhuman primate
NP: nucleoprotein
ORF: open reading frame
pre–MA-ZEBOV: precursor virus for generating mouse-adapted virus
VP: viral protein
WT-ZEBOV: wild-type virus
ZEBOV: *Zaire ebolavirus*
